# Deliberative Decision-Making in Macaques Removes Reward-Driven Response Vigor

**DOI:** 10.3389/fnbeh.2021.674169

**Published:** 2021-08-18

**Authors:** Nabil Daddaoua, Hank P. Jedema, Charles W. Bradberry

**Affiliations:** National Institute on Drug Abuse (NIDA) Intramural Research Program, Baltimore, MD, United States

**Keywords:** decision-making, choice, deliberation, movement vigor, addiction

## Abstract

Most of our daily decisions are governed by one of two systems: an impulsive system driving instantaneous decisions and a deliberative system driving thoughtful ones. The impulsive system reacts to immediately available concrete rewards. In contrast, the deliberative system reacts to more delayed rewards and/or punishments, which imposes consideration of longer-term choice consequences. Contingency management for addiction treatment is hypothesized to engage deliberative processes. Ultimately, in both decision-making situations, an action is needed to enact the decision. Whether those actions differ in implementation is an open question whose answer could inform as to whether distinct neural systems are engaged. To explore whether there is evidence of separate mechanisms between deliberated and immediate choices, we trained monkeys to perform a decision-making task where they made a choice on a touch screen between two visual cues predicting different amounts of reward. In immediate choice (IC) trials, the cues appeared at the final response locations where subjects could immediately touch the chosen cue. In deliberated choice (DC) trials, compound cues appeared orthogonally to the response locations. After a delay, allowing for decision formation, an identifying cue component was displaced to the randomly assigned response locations, permitting subjects to reach for the chosen cue. Both trial types showed an effect of cue value on cue selection time. However, only IC trials showed an effect of the competing cue on response vigor (measured by movement duration) and a reach trajectory that deviated in the direction of the competing cue, suggesting a decision reexamination process. Reward modulation of response vigor implicates dopaminergic mechanisms. In DC trials, reach trajectories revealed a commitment to the chosen choice target, and reach vigor was not modulated by the value of the competing cue. Our results suggest that choice–action dynamics are shaped by competing offers only during instantaneous, impulsive choice. After a deliberated decision, choice–action dynamics are unaffected by the alternative offer cue, demonstrating a commitment to the choice. The potential relevance to contingency management is discussed.

## Introduction

Recent decision-making theories posit that dynamics of enacting a decision are part of the decision-making process itself. Support for this comes from studies showing that, with immediate decisions, sensory evidence is accumulated across time prior to triggering an initial choice action. After action onset, subsequent sensory evidence can be used to confirm or possibly overrule the initial choice (Resulaj et al., 2009). These findings support a two-stage model of decision-making where post-decisional evidence can change the subject’s confidence in the selected option, possibly leading to a change of mind (Pleskac and Busemeyer, 2010; Murphy et al., 2015). In contrast to immediate decisions, others might be deliberated before enactment. Contingency management, a successful approach for addiction treatment (Higgins et al., 2004), is hypothesized to engage deliberative decision-making (Regier and Redish, 2015). In this paper, we asked the empirical question of whether a two-stage model of decision enactment applies both when consequences of choice between two options of differing reward value are available immediately and also when the animal is forced to deliberate its choice. To do so, we conducted comparisons between action dynamics of immediate and deliberated decisions that might implicate differential engagement of neural systems relevant to the efficacy of contingency management.

## Materials and Methods

### Subjects

Two adult male rhesus monkeys (*Macaca mulatta*), M1 and M2, were used in this study. Monkeys were housed in a temperature- and humidity-controlled vivarium that followed a 12-h light/dark cycle (light on at 7 a.m.). Animals were water regulated (30 ml/kg/day) in their home cage. Both animals contributed with 12 testing sessions (mean of 308 and 274 trials per sessions for M1 and M2). M1 was head fixed, allowing eye movement recordings. M1 contributed with 8 additional sessions (mean of 339 trials per session) in which hand movements were recorded (eye movements were not recorded in these behavioral sessions). All studies were approved by the NIDA-IRP Animal Care and Use Committee.

### Training Procedure

**FIGURE 1:**
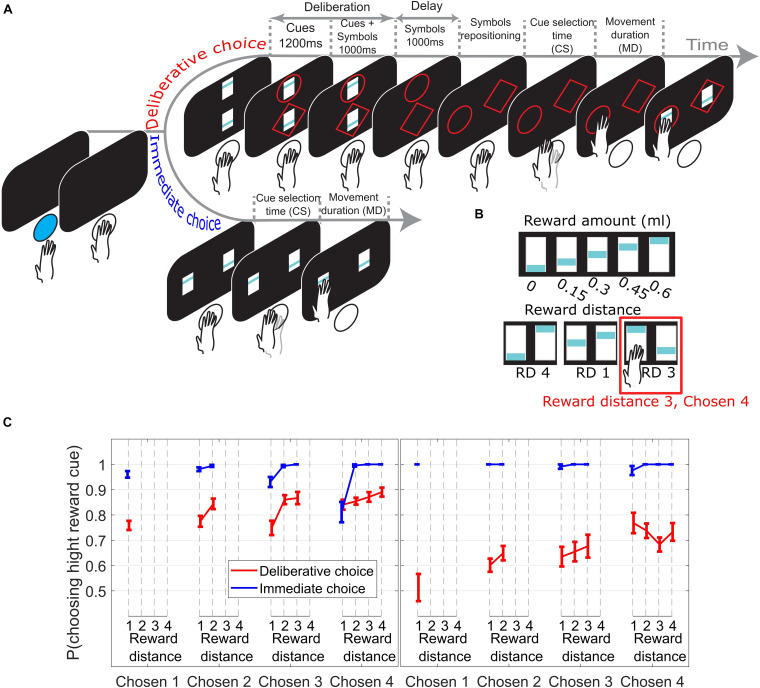
**(A)** Subjects initiated a trial by pushing a blue led button. In DC trials (top timeline) two reward cues appeared, with the offer magnitudes 0–4 units of juice reward signaled by the blue bar height. Next, a circle or a diamond (response-symbol) was randomly assigned and added to each reward cue. Reward cues were removed during the delay period. Next, the response-symbols where repositioned randomly to the response locations allowing the subjects to reach for the symbol corresponding to their reward choice. The reward cue corresponding to the chosen symbol was shown for an additional 300 ms as visual feedback and reward was delivered. In IC trials (bottom timeline), subjects reached for the chosen reward cue as soon as it appeared. **(B)** Upper row shows the reward cues used with their corresponding amount of juice reward. Lower row shows 3 example trials with the corresponding reward distance (RD) between cues. **(C)** Choice performance across sessions (± SEM) as function of reward distance between the choice offer cues for different Chosen Values. Left/right panel from M1/M2.

Monkeys were trained to perform a decision-making task where they chose between two visual cues predicting different amounts of juice rewards. Importantly, they expressed their choice by touching the chosen cue on a touch screen. The use of a non-ballistic choice action is crucial as it can reveal a decision reexamination process as the response unfolds (Song and Nakayama, 2009). In immediate choice (IC) trials (Figure 1A, bottom timeline), monkeys released the hold button and reached for the chosen reward cue as soon as it appeared. In deliberated choice (DC) trials (Figure 1A, top timeline), subjects had to hold the button for a 2,200 ms deliberation period while reward cues could be compared, and the associated response symbol could be remembered. After a 1,000-ms delay during which only response symbols were visible, these symbols were repositioned, signaling the monkeys to release the button and touch the symbol associated with their chosen reward. During the testing sessions, IC and DC trials were randomly interleaved.

Prior to collecting the data reported in this paper, monkeys were successively trained to perform three tasks. A forced choice task, to learn the reward cue values, followed by a choice task to check the subject’s understanding of the cue values, and finally a symbol association task. The trial structure of the forced choice task was very similar to IC trials. Briefly, in every trial, one reward cue was shown at one of the two possible response locations. The monkeys were allowed to release the hold button and reach for the reward cue as soon as it appeared in order to receive the corresponding amount of juice reward. After a few sessions, subjects were exposed to the choice task where the trial structure was identical to that in IC trials. Once the monkey’s choice probability of choosing a high reward cue reached 0.8, the monkeys were trained on the symbol association task. The trial structure of the symbol association task was very similar to the one in DC trials. The only difference being that only one reward cue was shown and associated with one response symbol. Briefly, during cue onset, only one reward cue was shown, after which two symbols were randomly assigned and added, one surrounding the reward cue (e.g., diamond) and the other symbol (e.g., circle) shown at the opposite side of the monitor (no other reward cue was shown at that location). After the delay period (initially set to 50 ms and subsequently increased to 1,000 ms), the symbols were repositioned to the response locations, and the monkeys were allowed to reach for one of the two symbols. If the monkeys chose to reach for the symbol previously associated with the reward cue (diamond in this hypothetical case), they received the juice reward corresponding to the reward cue associated with it. If they chose to reach for the symbol not associated with the reward cue (circle in this hypothetical case), they were not rewarded. Once the monkey’s choice probability of choosing the symbol associated with the reward cue reached 0.8, the monkeys were then tested on the final task (Figure 1A).

### Behavioral Measurements

Behavioral control was implemented in the NIMH MonkeyLogic (Hwang et al., 2019). Choice responses were collected using a touch screen (Elo TouchSystems; Menlo Park, CA).

In every trial, subjects were given a choice between two reward cues signaling different amounts of juice reward units (Figure 1B, upper row) ranging from 0 (0 ml juice) to 4 (0.6 ml juice) units of reward. Reward distance (RD) is defined as the difference between units of reward signaled by the cues (see Figure 1B, lower row for three examples). In this paper, we only consider correct trials (trials where the high reward cue was chosen). All the data reported here are means across testing sessions, and the error bars show the standard errors of the means.

**FIGURE 2:**
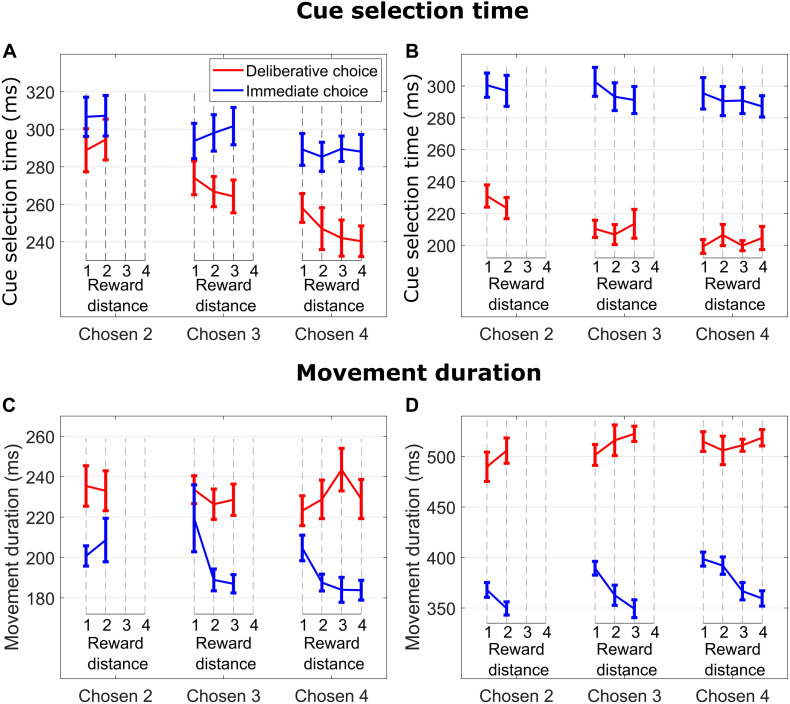
**(A)** Cue selection time (time from when the cues were presented to when the monkey released the hold button) vs. reward distance (RD) between cues for different Chosen Values for DC (red curves) and IC (blue curves) trials for M1. **(B)** Same format as in **(A)** for M2. **(C)** Movement duration (time from when the monkey released the hold button to when the monkey touched the reward cue) vs. reward distance (RD) between cues for different Chosen Value for DC (red curves) and IC (blue curves) trials for M1. **(D)** Same format as in **(C)** for M2.

**FIGURE 3:**
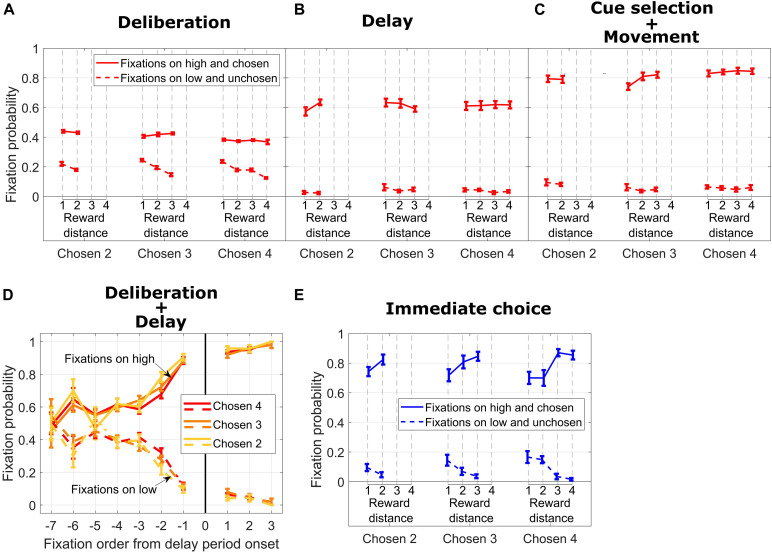
**(A)** Fixation probability on high/chosen (solid lines) and low/unchosen (dotted lines) cues vs. reward distance (RD) for different Chosen Values during DC trials, during the deliberation period. **(B)** Same format as in **(A)** for the delay period. **(C)** Same format as in B for cue selection and movement period. **(D)** Fixation probability on high/chosen (solid lines) and low/unchosen (dotted lines) cues for different Chosen Values vs. fixation order from end of the deliberation period. **(E)** Same format as in **(C)** for IC trials.

**FIGURE 4:**
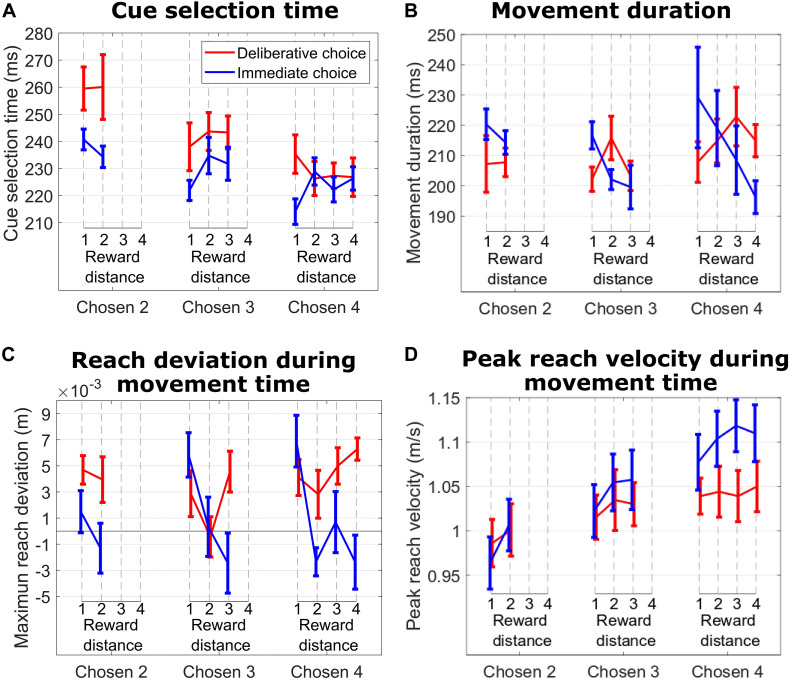
**(A)** Cue selection time vs. reward distance (RD) between cues for different Chosen Values for DC (red curves) and IC (blue curves) trials during the hand tracking sessions with subject M1. **(B)** Same format as in **(A)** for movement duration. **(C)** Same format as in B for maximum reach deviation. **(D)** Same format as in **(C)** for peak reach velocity.

Figure 1C presents choice performance from trials across all Chosen Values, broken down by RD. In Figures 2–4, only trials where subjects chose the high reward cue signaling 2, 3, or 4 reward units (Chosen Value 2, 3, or 4) were considered, as only these trials offer at least 2 data points to measure the effect of RD on the measured behavioral output (i.e., cue selection time and movement duration).

We measured 3D hand movements using an inertial measurement unit (NGIMU, x-io Technologies, United Kingdom) sampling at 240 Hz (Madgwick et al., 2011) attached to the monkey’s right wrist. In brief, gyroscope and accelerometer data were combined to compute rotational and translational wrist movement acceleration in a gravity-centered frame of reference. The 3D acceleration data were double integrated to get the 3D hand trajectories (Madgwick et al., 2011). Next, 3D hand reach trajectories to the left and right targets were projected onto a plane fitted to the raw data. With the resulting 2D data, we computed the maximum reach deviation distance between a straight line connecting the start and the end position and the actual hand trajectory for each trial type and RD. We defined the deviation as negative/positive when the trajectory was curved away/toward the unchosen cue.

We monitored eye position using an infrared system (ISCAN ETL-200) sampling at 240 Hz. For the analysis, the recorded horizontal and vertical eye position traces were smoothed with a Savitzky–Golay filter (window = 10 data points, polynomial degree = 4). The resulting filtered eye traces were subjected to a non-parametric algorithm using k-means cluster analysis to extract saccade reaction times and fixation locations (König and Buffalo, 2014).

## Results

### Reward Choice

On IC trials (Figure 1C, blue lines), subjects chose the high reward cue with the same high probability irrespective of the Chosen Value (linear regression of *p*(choose high cue) on Chosen Value collapsed across all RDs; M1: *p* = 0.17; M2: *p* = 0.24). In DC trials, however (Figure 1C, red lines), the probability of choosing the symbol associated with the high reward cue increased when the value increased (linear regression of *p*(choose high cue) on Chosen Value, collapsed across all RDs; M1: *p* = 1.3e−05; M2: *p* = 3.74e−07). The effect of RD on the probability of choosing the high reward cue was not consistent between the two monkeys on DC trials.

### Cue Selection Time

On IC trials, subjects quickly made a decision and selected the reward cue corresponding to their initial choice. At a given Chosen Value (2, 3, or 4), cue selection time (time from when the cues were presented to when the monkey released the hold button) was not modulated by RD (blue lines in Figure 2A for M1, all *p* > 0.55; blue lines in Figure 2B for M2, all *p* > 0.35), suggesting that choice initiation did not involve an ongoing consideration of the unchosen cue. On the other hand, cue selection time slightly decreased as Chosen Value increased. This effect was significant with subject M1 but did not reach significance level with subject M2 (linear regression of CS time on Chosen Value, collapsed across RDs; M1: *p* = 0.014; M2: *p* = 0.27), suggesting that choice action initiation was not consistently influenced by Chosen Value.

In DC trials, subjects had presumably enough time to make a decision during the 2,200-ms deliberation period. When the response symbols were randomly repositioned to the response locations, subjects searched and selected the response symbol (circle or diamond) corresponding to their chosen reward cue. As in IC trials, DC trials featured an absence of RD influence on cue selection time (red lines in Figure 2A for M1, all *p* > 0.15; red lines in Figure 2B for M2, all *p* > 0.43), suggesting again an absence of ongoing consideration of the unchosen cue when initiating the choice action. Cue selection time decreased as Chosen Value increased (linear regression of cue selection time on Chosen Value collapsed across all RDs; M1: *p* = 1.46e−07; M2: *p* = 2.49e−05).

The influence of Chosen Value on cue selection time was greater in DC then in IC trials. This effect was significant with subject M1 and was close to be significant with subject M2 (i.e., the cue selection time difference between Chosen Values 3 and 4 was greater in DC than in IC trials; linear regression of cue selection time on Chosen Value, with TrialType × Chosen Value interaction term, M1: *p* = 0.02; M2: *p* = 0.08). This suggests that cue selection was more sensitive to Chosen Value when the decision was deliberated, and expected reward value was known.

We also compared cue selection time on correct and error trials (Supplementary Figures 1A,B,E). Only trials where subjects chose a symbol associated with cues signaling 1, 2, or 3 reward units were included (trials highlighted with gray background in Supplementary Figure 1A), as only those trials offer data points from both trial types: Chosen Value 0 only offers data from error trials, whereas Chosen Value 4 only offers data from correct trials. For a given Chosen Value, the data were collapsed across all RDs. For both subjects (Supplementary Figure 1A for M1 and Supplementary Figure 1B for M2; Supplementary Figure 1E for M1 during the hand tracking sessions), cue selection time was shorter on error trials when subjects chose a symbol associated with cue signaling 1 unit of reward in most of the testing sessions (Wilcoxon rank sum test comparing medians of cue selection time on correct and error trials for Chosen Value 1; M1, *p* = 0.012; M2, *p* = 0.004; M1 hand tracking sessions, *p* = 0.1). Importantly, cue selection time was sensitive to Chosen Value only when subjects made a correct choice (linear regression of cue selection time on Chosen Value, collapsed across all RDs; M1: correct trials *p* = 0.0002, error trials *p* = 0.36; M2: correct trials *p* = 3.8e−05, error trials *p* = 0.36; M1 hand tracking sessions: correct trials *p* = 0.002, error trials *p* = 0.18). These findings suggest that choice errors made by subjects are due to a failure to deliberate or a failure to remember the symbol associated with the high reward cue.

Taken together, in both decision-making scenarios, cue selection time on a manual choice task seems to be insensitive to the value of the unchosen cue, suggesting that this period of time did not involve an ongoing cue comparison process. On the other hand, cue selection time was sensitive to the absolute value of the chosen cue. Taken together, the data so far suggest that choice initiation is compatible with literature showing reaction time sensitivity to the value of the reach target (Opris et al., 2011; Mosberger et al., 2016; Summerside et al., 2018).

### Movement Duration

There was an effect of RD on movement duration on IC trials when subjects chose and reached for cues signaling 3 and 4 reward units (blue lines in Figure 2C for M1, Chosen Value 4: *p* = 0.009; Chosen Value 3: *p* = 0.035; Chosen Value 2: *p* = 0.51; blue lines in Figure 2D for M2, Chosen Value 4: *p* = 0.0002; Chosen Value 3: *p* = 0.002; Chosen Value 2: *p* = 0.07). Specifically, subjects took more time to reach and touch a reward cue when the RD was low (hard decision), suggesting consideration of the unchosen cue throughout the reach movement period (see below data from hand movement tracking sessions).

In contrast, DC trials did not exhibit any influence of RD on movement duration when reaching for cues signaling 2, 3, or 4 reward units (red lines in Figure 2C for M1, all *p* > 0.44; red lines in Figure 2D for M2, all *p*-values > 0.2), nor did the value of the chosen cue (linear regression of movement duration on Chosen Value, collapsed across all RDs; M1 *p* = 0.75; M2 *p* = 0.17). These findings suggest an absence of consideration of the unchosen cue throughout the reach movement period when the choice was deliberated, indicating a commitment to the initial choice decision.

DC trials did not exhibit any influence of Chosen Value on movement duration on either correct or error trials (Supplementary Figures 1C,D for M1 and M2, respectively, Supplementary Figure 1F for M1 during the hand tracking sessions; linear regression of cue selection time on Chosen Value, collapsed across all RDs (M1: correct trials *p* = 0.47, error trials *p* = 0.52; M2: correct trials *p* = 0.09, error trials *p* = 0.36; M1 hand tracking sessions: correct trials *p* = 0.97, error trials *p* = 0.48). Given a Chosen Value of 1, 2, or 3, movement duration did not differ between correct and error trials (Wilcoxon rank sum test comparing medians of movement duration for Chosen Value 1, 2, or 3; M1, all *p* > 0.05; M2, all *p* > 0.05; M1 hand tracking sessions, all *p* > 0.05 except Chosen Value 2, *p* = 0.007).

Taken together, even though subjects made a choice to reach for a cue/symbol signaling the same amount of reward in both trial types, the cue selection process and the action implementing the choice differ in several respects. We show that, when subjects made an immediate decision (IC trials), cue selection time was insensitive to the unchosen cue value, but after the choice initiation, the value of the unchosen cue influenced movement duration, suggesting a choice revision process emerges in which the value of the competing cue either confirms or interferes with the initial decision. On the other hand, when subjects deliberated on the choice (DC trials), cue selection time was more sensitive to the target’s absolute value, but insensitive to the unchosen cue value.

### The Decision-Making Process

To what extent can we be sure that subjects came to a decision during the deliberation period on DC trials? Potentially, subjects could simply remember the values associated with each symbol (circle or diamond) and make their choice only after the go signal was given. To answer this, we analyzed eye movement data from subject M1. Figure 3A shows fixation probability on high (chosen) vs. low (unchosen) reward cues during the deliberation period (in Figure 1A, upper timeline, the deliberation period encompasses the “Cues” and “Cues + Symbols” periods of time). In general, fixation probability on the high and ultimately chosen reward cue was higher than fixation probability on the low and unchosen cue. When we sorted the trials according to Chosen Value, we did not find any significant effect of RD on fixation probability on high and chosen cues (solid red lines in Figure 3A, all *p* > 0.18). In contrast, fixation probability on low and unchosen cues decreased with increasing RD (dotted red lines in Figure 3A, Chosen Value 4: *p* = 2.04e−10; Chosen Value 3: *p* = 2.37e−08; Chosen Value 2: *p* = 0.01).

During the subsequent delay period, fixation probability on symbols corresponding to the high and chosen cues increased dramatically for all values of the chosen symbol and did not depend on RD (Figure 3B, solid lines, all *p* > 0.09). Fixation probability on the unchosen cue decreased nearly to zero, and the dependency on RD disappeared (Figure 3B, dotted lines, all *p* > 0.26). This pattern of fixations, in our opinion, signals a termination of the decision-making process. During this time frame, the subject also occasionally fixated on the response locations where the symbols were to be repositioned (data not shown).

Probability of fixation on the chosen cue increased even more during the subsequent time period encompassing cue selection and movement duration time periods and mostly were independent from RD (Figure 3C, solid lines, Chosen Value 4: *p* = 0.54; Chosen Value 3: *p* = 0.02; Chosen Value 2: *p* = 0.9). Also, fixation probability increased with Chosen Value (linear regression of fixation probability on Chosen Value, collapsed across all RDs; *p* = 0.004), showing a facilitation of selecting the high reward cue by the eye. Additionally, fixation probability on the unchosen cue stayed low and did not depend on RD (Figure 3C, dotted lines, all *p* > 0.5) nor on the absolute value of the chosen cue (linear regression of fixation rate on Chosen Value, collapsed across all RDs, *p* = 0.06).

To investigate the temporal aspect of fixation allocation during the deliberation period and the subsequent delay period, we calculated the probability of fixation on the chosen and unchosen reward cues (Figure 3D). As shown in Figure 3C, irrespective of Chosen Value, the subject fixated both cues with the same probability at the beginning of the deliberation period (from Fixations −7 to −5 in the x-axis in Figure 3D; Wilcoxon rank sum test comparing medians of fixation probability on high and low reward cues for a given Chosen Value and fixation order: Chosen Value 4, Fixation −7, *p* = 0.87; Fixation −6, *p* = 0.02; Fixation −5, *p* = 0.11; Chosen Value 3, Fixation −7, *p* = 0.63; Fixation −6, *p* = 0.03; Fixation −5, *p* = 0.17; Chosen Value 2, Fixation −7, *p* = 0.8; Fixation −6, *p* = 0.02; Fixation −5, *p* = 0.98). When considering the last four fixations during the deliberation period (Fixations −4 to −1), we found that the subject fixated the ultimately chosen reward cue more from Fixations −4 to −1 combined (Wilcoxon rank sum test comparing medians of fixation probability on high and low rewards: Chosen Value 4, *p* = 1.4e−08; Chosen Value 3, *p* = 3.09e−09; Chosen Value 2, *p* = 1.6e−08). This fixation bias increased sharply from Fixations −4 to −1 to reach a probability of fixating the high reward cue of 0.88 (Chosen Values 3 and 4) and 0.9 (Chosen Value 2). The first fixations on symbols associated with cues at the beginning of the delay period (Fixation 1 in the x-axis in Figure 3D) were predominantly on the symbol associated with the ultimately chosen high reward cue. This bias did not progress (from Fixations 1 to 3 in the x-axis in Figure 3D) as sharply as during the deliberation period, suggesting a commitment to choose the symbol associated with the high reward cue.

For IC trials, we considered all fixations on cues during a time window encompassing the cue selection and movement duration times (see Figure 1A lower timeline). As shown in Figure 3E, fixation probability on the high and ultimately chosen reward cue was higher than fixation probability on the low reward cue across all Chosen Values. Additionally, fixation probability on the ultimately chosen cue increased with RD for most of the Chosen Values (solid blue lines in Figure 3E, Chosen Value 4: *p* = 0.0005; Chosen Value 3: *p* = 0.0003; Chosen Value 2: *p* = 0.42). Fixation probability on the low unchosen reward cue decreased as RD increased for the higher Chosen Values (dotted blue lines in Figure 3E, Chosen Value 4: *p* = 0.0001; Chosen Value 3: *p* = 0.02; Chosen Value 2: *p* = 0.28). Taken together, when the subject faced an easy decision (high RD), fixation probability on the high reward cue was the highest and fixation probability on the low reward cue was the lowest. On the other hand, when the subject faced a hard decision (low RD), those two fixation probabilities were closer to each other, suggesting greater decision uncertainty requiring visual comparison.

### Choice Action Dynamics

To shed light on how the initial decision might be revised during choice action, we recorded 3D hand movements from subject M1 in separate sessions while performing the exact same task as in Figure 1A. The effects of RD and Chosen Value on cue selection time and movement duration largely replicated the effects seen in both monkeys without the wrist accelerometers and are summarized in Table 1.

**TABLE 1:** Upper table: *p*-values of linear regression of RD on CS time and MD for IC and DC trials (first rows) during the 3D hand tracking session.

		Cue selection time			Movement duration		
		Chosen4	Chosen3	Chosen2	Chosen4	Chosen3	Chosen2
**Immediate choice trials (IC)**	RD effect	0.17	0.23	0.26	0.04	0.03	0.36
	Chosen value effect	0.001			Regression not performed		
**Deliberative choice trials (DC)**	RD effect	0.38	0.6	0.9	0.37	0.89	0.96
	Chosen Value effect	7.9e–06			0.13		

		**Cue selection time vs. Chosen Value**					
**Trial type (IC vs. DC)**		0.03					

**p-values of RD chosen cue value on CS time and MD for IC and DC trials (2nd rows). Lower table: Linear regression of CS time on Chosen cue value, with TrialType X ChosenValue interaction term during the 3D hand tracking session, p = 0.03.**

The effect of RD on movement duration in IC trials suggested an emerging consideration of the unchosen cue as a possible choice target during the reach movement. This decision revision was absent when we analyzed the effect of RD on movement duration in DC trials, suggesting a commitment to the choice. Support for this interpretation comes from hand trajectory deviations and peak reach velocity. Specifically, during IC trials (Figure 4C, blue lines), we show that the subject’s hand trajectory deviated in the direction of the competing cue, and the amplitude of that deviation was greatest on trials with the smallest RD (hard trials). This effect was more pronounced when the subject reached for cues signaling 3 and 4 units of reward (Chosen Value 4: *p* = 0.017; Chosen Value 3: *p* = 0.014; Chosen Value 2: *p* = 0.31). When we considered DC trials (Figure 4C, red lines), the hand deviation was consistently positive (probably reflecting an idiosyncratic behavior), but importantly, this deviation did not depend on RD when the subject reached for cues signaling 2, 3, or 4 units of reward, suggesting an absence of consideration of the unchosen cue during the reach movement (all *p* > 0.19). The absolute value of the chosen target had a significant effect on peak reach velocity on IC trials, suggesting a motivational effect on choice action vigor (linear regression of peak velocity on Chosen Value, collapsed across all RDs; *p* = 0.01). This effect was absent on DC trials (*p* = 0.23).

## Discussion

We have shown that cue selection and choice action dynamics differ when the decision maker is forced to deliberate a decision prior to responding (DC), vs. enacting the choice response during decision formation (IC).

In IC trials, the unleashing of the initial decision did not involve an ongoing cue comparison process, as shown by the lack of influence of RD on cue selection time. However, after initiating the choice decision, IC trials revealed a continuing refinement of the initial choice throughout the post-decisional/action epoch. Specifically, reach duration was longer when subjects were confronted with a hard decision (low RD), suggesting a more pronounced competition between reward cues for selection. This competition was also reflected in the hand trajectory as it deviated more in the direction of the competing cue when the competition was harder to resolve. On top of the influence of the competing cue on action dynamics, the Chosen Value regulated the movement vigor (indexed here by peak reach velocity).

In DC trials, subjects made an initial, committed choice. As in IC trials, the unleashing of the decision did not involve an ongoing cue comparison process. After initiating the choice and in contrast to IC trials, DC trials revealed a continued absence of cue competition, as it took the subjects the same amount of time to reach and touch the symbol corresponding to the chosen reward irrespective of RD. This commitment to reach and touch the chosen symbol was also reflected by the absence of deviation in hand trajectory toward the competing symbol when subjects faced hard (low RD) or easy (high RD) decisions. Moreover, response vigor was not modulated by the expected value of the choice action.

In DC trials, the eye movement data during the deliberation and the subsequent delay periods revealed that the animal did indeed consider both choice options at the beginning of the deliberation. After a few fixations, the subject discarded the low reward cue as a possible choice. When the go signal was given, the eye and the hand selected the high reward symbol, and this cue selection for both effectors was modulated by the absolute value of the symbol corresponding to the chosen cue, but not by the value of the competing cue. Cue selection time was reduced on trials with greater Chosen Value (Figures 2A,B), while the eye selected the high cue with higher probability (Figure 3C). On the other hand, in IC trials, when the reward cues appeared, both effectors showed an effect of the value of the unchosen cue. More precisely, both fixation probability (Figure 3E) and hand movement duration (Figures 2C,D) depended on RD. These data are compatible with literature (Khan et al., 2011; Nissens and Fiehler, 2018) showing a shared single attentional resource between the hand and the eye when planning a visually guided reach movement. Thus, on IC trials, the attentional drive continued to extract information pertinent to the choice in a way that gave the unchosen cue influence over action dynamics. In DC trials, attention deployed to select the high reward cue without influence of the unchosen cue.

In both trial types, cue selection time depended on the absolute value of the chosen cue. On IC trials, movement duration depended on RD and Chosen Value, whereas on DC trials, it did not depend on either. This dissociation of the influence of the decision-making context on cue selection time and movement duration has been reported with human subjects (Wong et al., 2015), suggesting that cue selection time and movement duration are independent movement parameters.

The observed difference in choice action vigor regulation for the two decision-making situations is indicative of the involvement of separate decisional systems. More precisely, it has been shown that decisions involving an immediately available reward are associated with activation of the midbrain dopamine system (McClure et al., 2004), which has also been shown to causally regulate action vigor (Niv et al., 2007; Beierholm et al., 2013; da Silva et al., 2018; Mendonça et al., 2021). Thus, we believe that our IC trials unleash an impulsive decisional system (Dayan et al., 2006) that incorporates neural systems purported to support the ability of drug cues to trigger craving and relapse (Phillips et al., 2003). On the other hand, the lateral prefrontal cortex has been shown to support decisions yielding a more distal and abstract reward (McClure et al., 2004). Therein, the predicted value of the chosen option (which was reflected in cue selection time in DC trials) was causally encoded (Lak et al., 2020). This brain region has also been shown to support imagination and evaluation of future outcomes, two important attributes of deliberation (Schacter et al., 2017).

In addiction, an impulsive decisional system, including dopaminergic elements, is believed to drive behavior (Volkow et al., 2008, 2017). Contingency management, an effective treatment for drug addiction (Higgins et al., 2004), is thought to temper this behavioral drive by providing an alternative reinforcer (money) that forces subjects into a deliberative mode (Regier and Redish, 2015). In light of our findings, we speculate that deliberation engaged by contingency management helps subjects to make decisions based on a considered evaluation of options: drug taking with negative consequences vs. money. Importantly, as we show here, those deliberated decisions appear to disengage dopaminergic systems known to modulate reward-driven vigor and that are believed to support drug craving.

## Data Availability Statement

The original contributions presented in the study are included in the article/Supplementary Material, further inquiries can be directed to the corresponding author/s.

## Ethics Statement

The animal study was reviewed and approved by the NIDA-IRP Animal Care and Use Committee.

## Author Contributions

ND, HJ, and CB designed the research and wrote the manuscript. ND performed the research. ND and HJ analyzed the data. All authors contributed to the article and approved the submitted version.

## Conflict of Interest

The authors declare that the research was conducted in the absence of any commercial or financial relationships that could be construed as a potential conflict of interest.

## Publisher’s Note

All claims expressed in this article are solely those of the authors and do not necessarily represent those of their affiliated organizations, or those of the publisher, the editors and the reviewers. Any product that may be evaluated in this article, or claim that may be made by its manufacturer, is not guaranteed or endorsed by the publisher.

## Supplementary Material

Supplementary Figure 1 **(A)** Cue selection time vs. Chosen Value in DC trials for correct (dark red curves) and error (light red curves) trials during the main data set sessions with subject M1. **(B)** Same format as in **(A)** for subject M2. **(C)** Same format as in **(A)** for movement duration for subject M1. **(D)** Same format as in **(C)** for subject M2. **(E)** Same format as in **(A)** during the hand tracking sessions with subject M1. **(F)** Same format as in C during the hand tracking sessions with subject M1. The trials highlighted with shaded area in A were used to compute the effect of the chosen cue value on cue selection time and movement duration.
